# A Five-MicroRNA Signature Predicts the Prognosis in Nasopharyngeal Carcinoma

**DOI:** 10.3389/fonc.2021.723362

**Published:** 2021-09-09

**Authors:** Shixiong Wu, Cen Zhang, Jing Xie, Shuang Li, Shuo Huang

**Affiliations:** Department of Otolaryngology, Zhongnan Hospital of Wuhan University, Wuhan, China

**Keywords:** nasopharyngeal carcinoma, microRNA, prognosis, chemotherapy response, predicts

## Abstract

**Background:**

There is no effective prognostic signature that could predict the prognosis of nasopharyngeal carcinoma (NPC).

**Methods:**

We constructed a prognostic signature based on five microRNAs using random forest and Least Absolute Shrinkage And Selection Operator (LASSO) algorithm on the GSE32960 cohort (*N* = 213). We verified its prognostic value using three independent external validation cohorts (GSE36682, *N* = 62; GSE70970, *N* = 246; and TCGA-HNSC, *N* = 523). Through principal component analysis, receiver operating characteristic curve analysis, and C-index calculation, we confirmed the predictive accuracy of this prognostic signature.

**Results:**

We calculated the risk score based on the LASSO algorithm and divided the patients into high- and low-risk groups according to the calculated optimal cutoff value. The patients in the high-risk group tended to have a worse prognosis outcome and chemotherapy response. The time-dependent receiver operating characteristic curve showed that the 1-year overall survival rate of the five-microRNA signature had an area under the curve of more than 0.83. A functional annotation analysis of the five-microRNA signature showed that the patients in the high-risk group were usually accompanied by activation of DNA repair and MYC-target pathways, while the patients in the low-risk group had higher immune-related pathway signals.

**Conclusions:**

We constructed a five-microRNA prognostic signature, which could accurately predict the prognosis of nasopharyngeal carcinoma, and constructed a nomogram that could conveniently predict the overall survival of patients.

## Introduction

Nasopharyngeal cancer (NPC) is a rare tumor, with the highest incidence in southeast Asia, such as southeast China, India, and Thailand (1). The main cause of treatment failure in NPC patients was the occurrence of local metastatic events, in which more than 70% of patients presented local progression (2). An accurate prognosis prediction can guide the selection of treatment options for patients with NPC. Currently, the tumor lymph node metastasis (TNM) system is still a widely recognized system for predicting prognosis (3). However, previous studies have found that, among NPC patients with the same tumor stage, even after receiving similar treatment regimens, the treatment outcomes are quite different (4). The current TNM staging system only focuses on the anatomical features of the tumor and ignores the biological heterogeneity of the tumor so that it cannot accurately predict the prognosis of individual patients (5). Therefore, it is urgent to find a new biomarker that could predict the prognosis independently or in combination with the TNM staging system so as to improve the accuracy of the prediction.

MicroRNA plays a significant role in the development of cancer and its therapeutic response. Recent studies have demonstrated the value of some microRNA in predicting the prognosis of NPC, and some microRNA signatures have been constructed to predict the prognosis of NPC (6–8). Studies have reported that Epstein–Barr virus (EBV)-related microRNA have potentially important effects on NPC. The in-depth study of microRNA encoded by EBV has potential significance and importance for the discovery of prognostic markers of NPC (9).

At present, Least Absolute Shrinkage and Selection Operator (LASSO) has been widely used to construct the prognostic model (10–12). Here we developed and validated a prognostic signature based on five microRNAs in four large cohorts (GSE32960, GSE36682, GSE70970, and TCGA-HNSC)—a total of 1,143 patients with NPC. The random forest and LASSO Cox regression algorithm were used to select the characteristics and quantify the Cox regression coefficient, to build a scoring model, which could not only accurately predict the prognosis and progress of NPC. An accurate nomogram was provided for clinicians to predict the prognosis of NPC patients.

## Materials and Methods

### Cohort Information and Data Preprocessing

**Figure 1:**
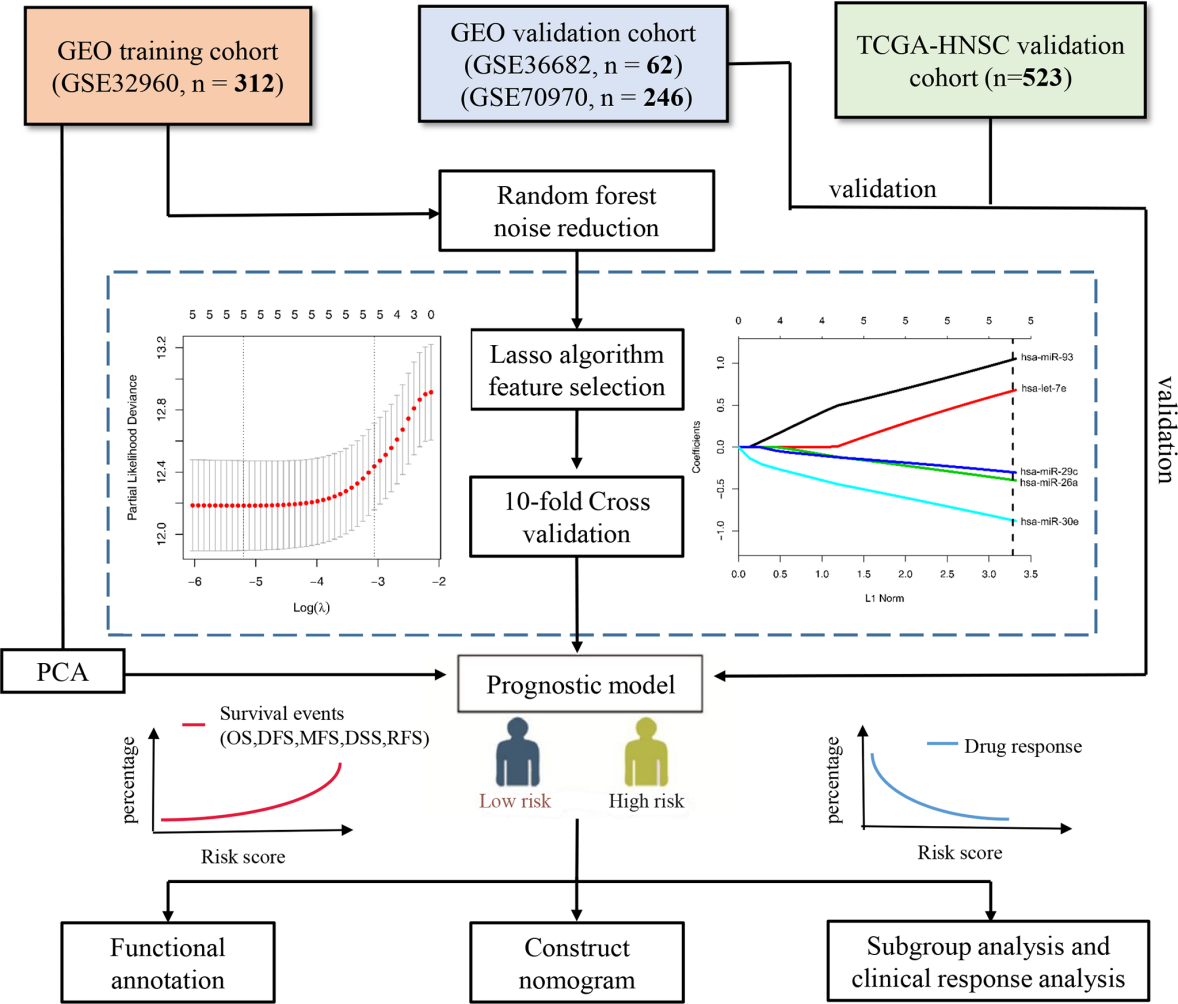
Flow chart of the study. PCA, principal component analysis; OS, overall survival; DFS, disease-free survival; MFS, metastasis-free survival; DSS, disease-free survival; RFS, relapse-free survival.

We selected four large microRNA microarray/sequencing cohorts (GSE32960: *N* = 312, GSE36682: *N* = 62, GSE70976 (13): *N* = 246, and TCGA-HNSC: *N* = 523) from the Gene Expression Omnibus (GEO; https://www.ncbi.nlm.nih.gov/geo/) and The Cancer Genome Atlas (TCGA; https://cancergenome.nih.gov/) database, all of which contained detailed prognostic follow-up data. The cohort (GSE32960) with the largest number of patients in the GEO database was selected as the training cohort, and the other three cohorts (GSE36682, GSE70976, and TCGA-HNSC) were selected as independent external validation cohorts. The GSE32960 cohort contained 312 non-metastatic NPC tissues and 18 non-cancerous nasopharyngitis tissues. The GSE36682 cohort contained 62 cases of NPC and six cases of normal tissues. The GSE70976 cohort contained 246 cases of NPC and 17 cases of normal tissues. We retained only NPC samples with prognostic follow-up data. The level 3 microRNA sequencing data of head and neck squamous cell carcinomas were downloaded from the UCSC Xena website (https://gdc.xenahubs.net/download/TCGA-HNSC.mirna.tsv.gz) based on the TCGA database. The cohort contained 523 head and neck squamous cell carcinoma samples (including NPC). The data normalization method adopted reads per million mapped reads. All data were log2(*x* + 1) transformed. The baseline information for all cohorts used in this study is listed in **Table 1**, and the flow chart of the experiment is shown in **Figure 1**.

**Table 1:** Baseline information for the cohort that we used.

Series accession numbers	GSE32960	GSE36682	GSE70970	TCGA-HNSC
Platform	MicroRNA array	Human miRNA 1K	nCounter^®^ Human miRNA assay (v1.0, Nanostring)	Illumina miRNAseq
Number of patients	312	62	246	523
T				
Tx	–	–	2	12
T1	66	–	74	38
T2	89	–	50	149
T3	71	–	52	140
T4	86	–	67	184
N				
Nx	–	–	1	19
N0	44	–	49	245
N1	148	–	83	85
N2	72	–	90	165
N3	48	–	23	9
M				
Mx	–	–	–	20
M0	–	–	–	497
M1	–	–	–	6
Stage				
I	12	–	–	27
II	86	–	–	84
III	91	–	–	92
IV	123	–	–	320
Age				
≥65	–	26	35	196
<65	–	36	211	326
Chemotherapy				
Yes	268	–	126	169
No	44	–	120	342
Unknown/not available	–	–	–	17
Survival outcome	Overall survival (OS), metastasis-free survival (MFS), disease-free survival (DFS), relapse-free survival (RFS)	OS	OS, MFS, DFS, RFS, DSS	OS, MFS

### Construct and Validate the Five-MicroRNA Signature

We first used the random forest algorithm to feature select all microRNA and found the microRNA which had the confirmed ability to distinguish the overall survival outcome (dead *vs*. alive). Next, we performed the LASSO Cox regression analysis using the R package “glmnet” (14) on training cohort GSE32960. The LASSO algorithm can reduce the dimensionality of high-latitude data and explain the characteristics of the data with a model with fewer variables (15). Tenfold cross-validation was used to avoid overfitting the model built with the training cohort. Finally, we constructed a scoring system based on the regression coefficients calculated by the LASSO cox regression analysis. We divided all NPC patients into high- and low-risk groups according to the cutoff values given by R package “survminer”. The Kaplan–Meier survival curve was used to explore the prognostic value of the five-microRNA signature, the time-dependent receiver operating characteristic (ROC) curve was used to measure the prediction accuracy, and principal component analysis (PCA) was used to observe the ability of the model to distinguish the overall survival outcome events. The prognostic value of the five microRNAs was validated by three independent external validation cohorts (GSE36682, GSE70970, and TCGA-HNSC).

### Functional Annotation of the Five-MicroRNA Signature

To carry out functional annotation with the five-microRNA signature, we downloaded RNA-seq data from the TCGA database. By calculating the correlation coefficient between risk score and all genes, the top 500 genes with the highest correlation were extracted for Gene Ontology, Kyoto Encyclopedia of Genes and Genomes, and gene set enrichment analysis (GSEA). Enrichment analysis and visualization were performed by the R package “ClusterProfiler” (16).

### Statistical Analysis

For the statistical test of the two groups of data, if the data characteristics meet the normal distribution, the *t*-test was used; if the data characteristics do not meet the normal distribution, the non-parametric test (Wilcoxon test) was chosen. The R package “rms” was used to construct a nomogram. The R package “rmda” was used to perform decision curve analysis (DCA) to evaluate the net benefits of the prediction signature (17). *P*-value < 0.05 was considered statistically significant.

## Results

### Feature Selection and Prognostic Signature Construction

We used random forest algorithm to select the features of all microRNAs in the training cohort GSE32960. Random forest is an algorithm that can effectively reduce the dimension and noise of high-latitude data (18). After selection, only five microRNAs (has-miR-93, has-let-7e, has-miR29c, has-miR-26a, and has-miR-30e) were selected as candidate features. Subsequently, we used the LASSO Cox regression algorithm to construct a prognostic signature based on the five microRNAs (**Figure 1**). The calculation method of the scoring model is as follows: risk score = 1.088 * hsa-miR-93 + 0.673 * hsa-let-7e + (-0.299) * hsa-miR-29c + (-0.395) * hsa-miR-26a + (-0.927) * hsa-miR-30e. The gene symbols of microRNA in the formula represent the transcriptional expression of the microRNA.

### The Prognostic Value of the Five-MicroRNA Signature Was Validated in Multiple Cohorts

**Figure 2:**
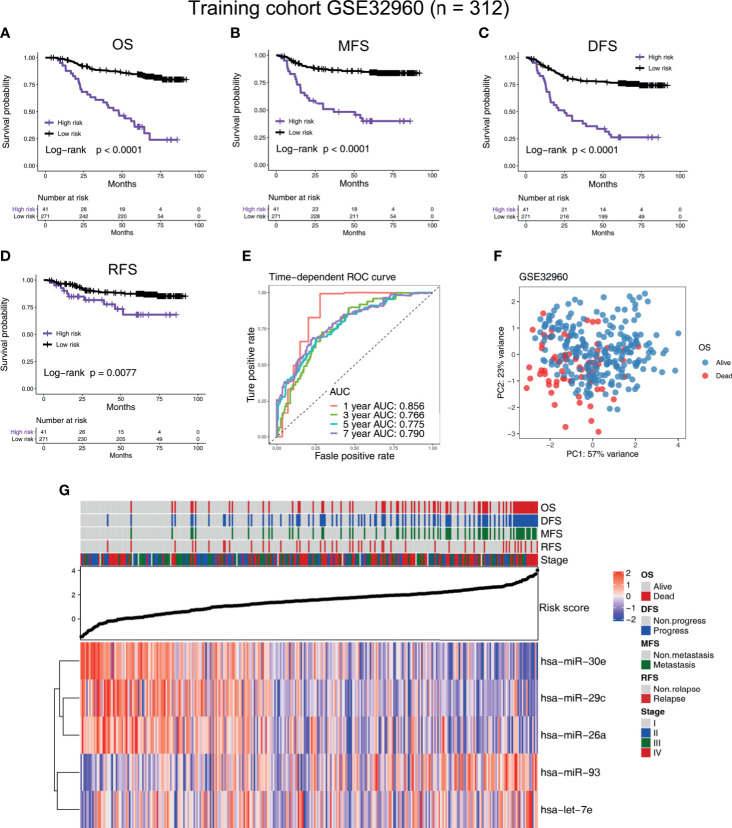
Construction of the five-microRNA prognostic signature on a training cohort (GSE32960). Kaplan–Meier survival curves of **(A)** overall survival, **(B)** metastasis-free survival, **(C)** disease-free survival, and **(D)** relapse-free survival in different risk groups in the training cohort (GSE32960). **(E)** The time-dependent receiver operating characteristic curve was used to assess the prognostic accuracy. **(F)** Principal component analysis was used to investigate the ability of five-microRNA to distinguish prognostic outcomes. **(G)** Heat map of the expression of five-microRNA in the training cohort GSE32960. The dot plot represents the value of the risk score. The red square in the heat map represents the high expression level of the microRNA, while the blue represents the low expression level.

**Figure 3:**
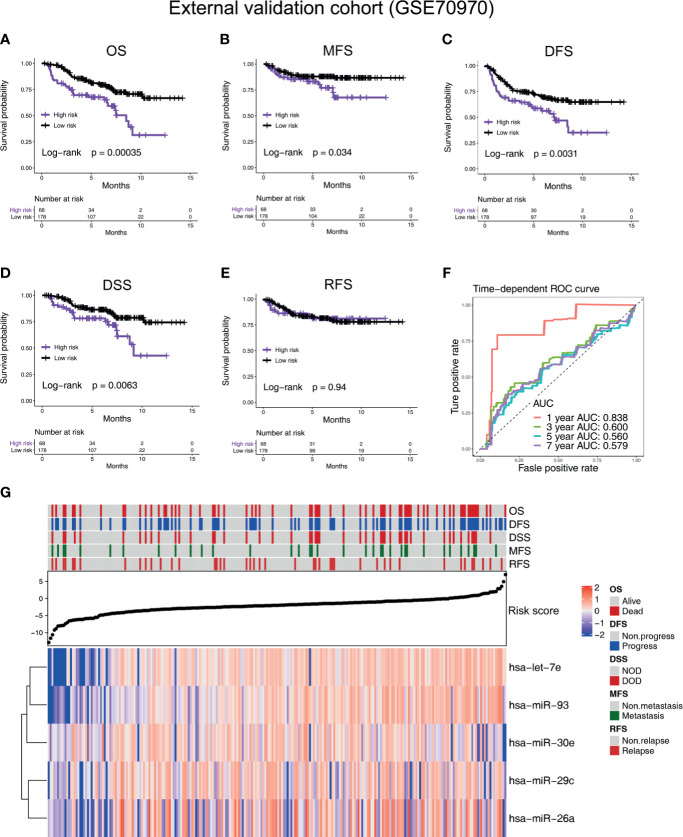
Verifying the prognostic value of the five-microRNA prognostic signature in an external validation cohort (GSE70970). Kaplan–Meier survival curves of **(A)** overall survival, **(B)** metastasis-free survival, **(C)** disease-free survival, **(D)** DSS, and **(E)** relapse-free survival in different risk groups in the external validation cohort (GSE70970). **(F)** The time-dependent receiver operating characteristic curve was used to assess the prognostic accuracy. **(G)** Heat map of the expression of the five-microRNA in the external validation cohort (GSE70970). The dot plot represents the value of the risk score.

According to the cutoff value given by R package “survminer” (19), the NPC patients in all the cohorts were divided into high-risk and low-risk groups. In the training cohort GSE32960, our results indicate that the five-microRNA signature has a high predictive value for overall survival (OS; hazard ratio, HR = 5.54; 95% confidence interval, CI: 2.58–11.89, *p* < 0.0001), metastasis-free survival (MFS; HR = 4.91, 95%CI: 2.23–10.81, *p* < 0.0001), disease-free survival (DFS; HR = 4.24, 95%CI: 2.16–8.33, *p* < 0.0001), and relapse-free survival (RFS; HR = 2.52, 95%CI: 0.96–6.61, *p* = 0.0077) of NPC (**Figures 2A–D**). In the external validation cohort GSE36682, we found that the five-microRNA signature has a high predictive value for the OS (HR = 2.87, 95%CI: 1.24–6.63, *p* = 0.0299) of NPC (**Supplementary Figure S1A**). In the external validation cohort GSE70970, we found that the five-microRNA signature has a high predictive value for OS (HR = 2.32, 95%CI: 1.33–4.03, *p* = 0.0003), MFS (HR = 2.05, 95%CI: 0.95–4.42, *p* = 0.0337), DFS (HR = 1.90, 95%CI: 1.16–3.10, *p* = 0.0031), disease-specific survival (DSS, HR = 2.19, 95%CI: 1.12–4.28, *p* = 0.0063), and RFS (HR = 2.19, 95%CI: 1.10–4.36, *p* = 0.9449; **Figures 3A–E**). The TCGA-HNSC cohort showed that the prognostic signature could predict the OS (HR = 1.57, 95%CI: 1.15–2.14, *p* = 0.0013) and MFS (HR = 1.68, 95%CI: 1.15–2.43, *p* = 0.0024) of NPC (**Supplementary Figures S2A, B**). The time-dependent ROC curves in the GSE32960, GSE36682, GSE70970, and TCGA-HNSC cohorts showed that the five-microRNA signature had a high prediction accuracy. In particular, the area under the curve of prediction of 1-year OS was greater than 0.83 in three cohorts (GSE32960: 0.856, **Figure 2E**; GSE70970: 0.838, **Figure 3F**; and GSE36682: 0.898, **Figure S1B**). The PCA showed that the dominant features of the five microRNAs could significantly distinguish the prognostic outcome of OS (**Figure 2F**). The heat map of the expression of the five microRNAs indicated that, with the increase of risk score, each microRNA has a significant trend of change, and the higher the risk score is, the more survival outcome events will occur (**Figures 2G**, **3G**, **Supplementary Figures S1C**, and **S2**). We calculated the C-index of the prognostic model in all the cohorts and found that the C-index in all of the three cohorts of GEO was greater than 0.77, indicating the high consistency of the prediction model (**Supplementary Table S3**).

### The Nomogram Was Constructed by Cox Regression Analysis

**Figure 4:**
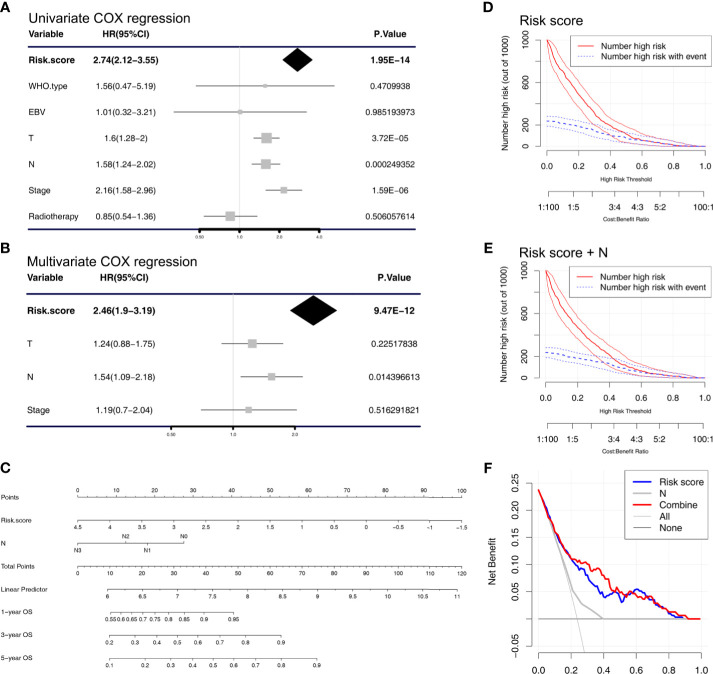
Cox regression analysis and establishment of the nomogram. **(A)** Univariate and **(B)** multivariate COX regression analyses were performed in the training cohort (GSE32960). **(C)** A nomogram that predicts the 1-, 3-, and 5-year overall survival of nasopharyngeal carcinoma patients. Clinical impact curve of the **(D)** risk score model and the **(E)** combined risk score and N stage model. The red curve (number of high risk) represents the number of people classified as positive (high risk) by the risk score model **(D)** or combined risk score and N stage model **(E)** under each threshold probability. The blue curve (number of high risk with outcome) is the number of people who are truly positive under each threshold probability. **(F)** Decision curve analysis was used to evaluate the clinical utility of the nomogram.

We used univariate and multivariate Cox regression analysis to analyze some prognostic factors, including risk score, in the training cohort GSE32960 and found that risk score (HR = 2.46, 95%CI: 1.90–3.19, *p* < 0.001) and N stage (HR = 1.54, 95%CI: 1.09–2.04, *p* = 0.0144) could predict OS in NPC patients independently of other factors (**Figures 4A, B**). Based on this result, we constructed a nomogram that conveniently predicted the OS of each NPC patient for 1, 3, and 5 years (**Figure 4C**). We found through DCA and clinical impact curve that the risk score combined with N stage could improve the clinical net benefits (**Figures 4D–F**).

### Subgroup Survival Analysis and Functional Annotation of the Five-MicroRNA Signature

**Figure 5:**
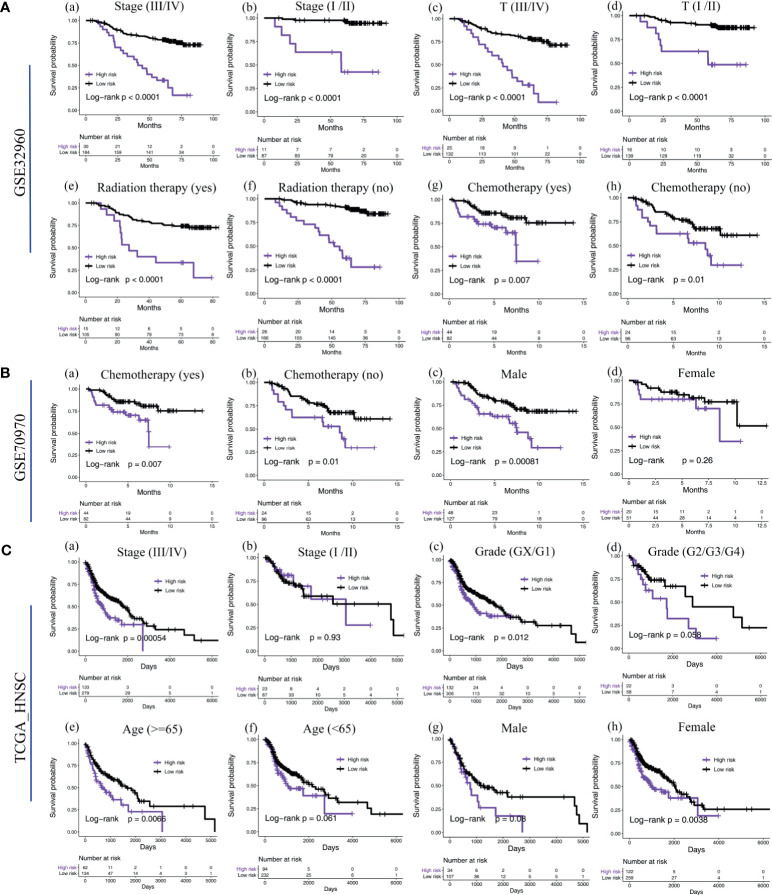
Subgroup survival analysis of the five-microRNA signature. **(A)** Subgroup survival analysis of the five-microRNA signature in the training cohort GSE32960. Including different stages (a, b), T stages (c, d), whether radiotherapy (e, f), and chemotherapy (g, h). **(B)** Subgroup survival analysis of the five-microRNA signature in the external validation cohort GSE70970. Including whether chemotherapy (a, b) and different gender (c, d). **(C)** Subgroup survival analysis of the five-microRNA signature in the external validation cohort TCGA-HNSC. Including different stages (a, b), grades (c, d), age (e, f), and gender (g, h).

We found that, in NPC patients with different tumor stages (III/IV *vs*. I/II), T stages (III/IV *vs*. I/II), gender (male *vs*, female), age (>=65 *vs*. <65), whether or not to undergo radiotherapy and whether or not to undergo chemotherapy, the five-microRNA signature showed a remarkable ability to predict OS (**Figure 5**). In the TCGA head and neck squamous cell carcinoma cohort, a subgroup analysis based on anatomical site subdivision showed a little difference in the expression of the risk score in each site (**Supplementary Figure S3**).

**Figure 6:**
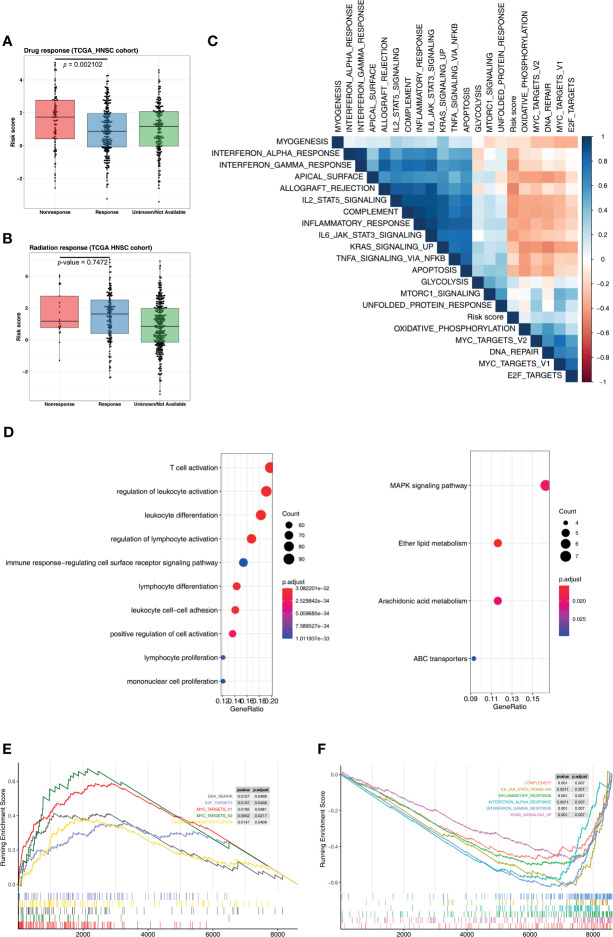
Five-microRNA signature predicts the chemotherapy response and functional annotation. Distribution of risk scores in different **(A)** chemotherapy and **(B)** radiotherapy responses. **(C)** Heat map showing the correlation between the risk score and the signaling pathways. The blue square represents a positive correlation, and the red square represents a negative correlation. **(D)** Gene Ontology and Kyoto Encyclopedia of Genes and Genomes analysis for the five-microRNA signature. Enrichment plots of gene set enrichment analysis based on the risk score, the pathway activated by the **(E)** high and **(F)** low risk score.

To understand the signaling pathways and biological functions associated with the five-microRNA signature, we conducted an enrichment analysis of the 500 genes most associated with the risk score, and the results indicated that the five-microRNA signature was associated with T cell activation, B cell activation, regulation of T cell activation, regulation of lymphocyte activation, immune response, and other biological functions (**Figure 6D** and **Supplementary Table S1**) as well as associated with MAPK, ether lipid metabolism, arachidonic acid metabolism, and ABC transporter, and other signaling pathways (**Figure 6D** and **Supplementary Table S2**). The GSEA analysis results indicated that patients with a high-risk score were usually activated by DNA repair, MYC targets, E2F targets, mTORC1, oxidative phosphorylation, and other signaling pathways (**Figure 6E**, **Table 2**). The NPC patients in the low-risk group were usually accompanied by a higher activation of INF-γ, INF-α, KRAS, inflammatory response, apoptosis, and other related signaling pathways (**Figure 6F** and **Table 2**). The risk score was significantly correlated with the signal values of these signaling pathways (**Figure 6C**).

**Table 2:** Gene set enrichment analysis of the risk score.

Term	Set size	Enrichment score	NES	*P*-value	Rank
MYC_TARGETS_V2	28	0.67	3.95	0.00	2,139
DNA_REPAIR	76	0.42	3.40	0.01	1,096
OXIDATIVE_PHOSPHORYLATION	94	0.39	3.30	0.02	2,006
E2F_TARGETS	86	0.36	3.05	0.01	3,234
GLYCOLYSIS	85	0.23	1.99	0.01	3,083
MTORC1_SIGNALING	95	0.23	1.95	0.02	2,118
UNFOLDED_PROTEIN_RESPONSE	54	0.25	1.84	0.01	460
MYOGENESIS	125	-0.30	-1.73	0.00	4,863
TNFA_SIGNALING_VIA_NFKB	106	-0.33	-1.81	0.00	3,089
APICAL_SURFACE	21	-0.49	-1.89	0.00	2,934
APOPTOSIS	79	-0.36	-1.90	0.00	2,500
IL2_STAT5_SIGNALING	116	-0.46	-2.55	0.00	1,478
COMPLEMENT	122	-0.47	-2.66	0.00	2,436
KRAS_SIGNALING_UP	120	-0.48	-2.71	0.00	1,238
INFLAMMATORY_RESPONSE	134	-0.50	-2.89	0.00	2,565
IL6_JAK_STAT3_SIGNALING	60	-0.62	-3.13	0.00	1,459
INTERFERON_ALPHA_RESPONSE	85	-0.59	-3.14	0.00	1,682
INTERFERON_GAMMA_RESPONSE	163	-0.62	-3.68	0.00	1,964
ALLOGRAFT_REJECTION	147	-0.70	-4.08	0.00	987

NES, normalized enrichment score.

Next, the R package “miRNAtap” was used for functional enrichment analysis of each microRNA separately (has-miR-93, has-let-7e, has-miR29c, has-miR-26a, and has-miR-30e). It was found that each microRNA participated in different major biological functions, such as hsa-let-7e—which was mainly involved in cell cycle-related signaling pathways (cell cycle process, DNA replication, regulation of cell cycle process, cell cycle phase transition, *etc.*), hsa-miR-29c—which was mainly involved in the regulation of gene expression-related signaling pathways, and hsa-miR-30e—which was mainly involved in the regulation of metabolic-related biological events (carboxylic acid metabolic process, coenzyme metabolic process, ribonucleotide metabolic process, *etc.*; **Supplementary Table S4**).

### Five-MicroRNA Signature Is Associated With Chemotherapy Response and Immune Checkpoint

An analysis of the clinical efficacy of the TCGA-HNSC cohort exhibited that, among NPC patients undergoing chemotherapy, the risk score of nonresponse patients was significantly higher than that of patients with response (*p* = 0.0021), indicating that the five-microRNA signature is a potential prediction target of chemotherapy (**Figure 6A**). At the same time, we found that the risk score had no predictive value for the radiotherapy response (*p* = 0.7472, **Figure 6B**). By calculating correlations between risk scores and major immune checkpoints, we found that the risk scores were significantly negatively correlated with PD-L1 and PD-1, but not with TMB (**Supplementary Figure S4**). Moreover, the risk score was also correlated with tumor stage and T stage, while N stage was not (**Supplementary Figure S4**).

## Discussion

NPC is a kind of malignant head and neck cancer (20, 21). At present, the development of comprehensive treatment methods of radiotherapy and chemotherapy has significantly improved the prognosis and outcome of NPC patients. However, NPC is still considered a malignant and invasive tumor due to its high aggressiveness, delayed diagnosis, and relatively poor prognosis (22).

MicroRNAs are endogenous non-coding RNA families of approximately 22 nucleotides in length (23). miRNAs are believed to have important effects on the development, progression, and response to treatment of tumors (24). Currently, researchers have found that microRNA plays a crucial role in the development of NPC. In recent years, studies on the pathogenesis and development of microRNA in NPC and its prognosis have been developing rapidly (9, 25–28). A meta-analysis identified 65 microRNAs as potential prognostic markers for NPC, providing new targets for future studies (29).

Currently, there are some prognostic signatures of microRNA in NPC (6, 7, 30, 31). Two miRNAs in the five-miRNA signature, has-miR29c and has-miR-30e, have been studied in nasopharyngeal carcinoma (32–34). It is possible that these two miRNAs have a particularly significant predictive role in the prognosis of nasopharyngeal carcinoma, so they are also used in other signatures. However, most of these studies ignore the analysis of subgroups and cannot prove that the prognostic signature still has a predictive value in certain subgroups of NPC patients. Our study conducted a subgroup analysis on the prognostic signature of five microRNAs and found that the five-microRNA signature showed a good prognostic value in each subgroup (different stages, grades, whether chemotherapy or radiotherapy, *etc.*), indicating that the five-microRNA signature could accurately predict the prognosis of various groups of NPC patients. At the same time, our study added a large number of cohorts to verify the results so as to avoid the overfitting effect and ensure that the model can exert its efficiency stably.

Resistance to chemotherapy is currently a major concern in the treatment of NPC. Recently, many studies have found that microRNAs are key molecules in the production of resistance to chemotherapy (35, 36). Our results indicated that the risk score based on the five-microRNA signature was negatively correlated with the efficacy of chemotherapy. We could predict the chemotherapy sensitivity of NPC patients according to the risk score so as to propose specific treatment plans for each NPC patient. However, due to the small number of cohorts with documented chemotherapy responses, our results were not validated in more cohorts. We will collect our chemotherapy response data in the future to verify our conclusions.

Through the analysis of the function annotation, we found that the higher risk score tends to the activation of cell cycle-related signal pathways, and the low-risk group of patients with significant immune response related to activating signaling pathways; it also provides a new way of thinking for the treatment of NPC patients, namely, the high-risk group of patients can try drugs with a targeted cell cycle, and the low patriarch of patients can try immune checkpoint inhibitors. This part of the conjecture needs further verification, which is our future research direction.

In addition, according to the univariate and multivariate Cox regression analyses, we found that the risk score and N stage could be independent of other prognostic factors to predict the prognosis of patients with NPC. On this basis, we constructed a nomogram, which could be convenient to predict the 1, 3, and 5-year overall survival in patients with NPC. The DCA analysis found that the joint risk score and N stage could have a higher clinical net benefit.

In summary, through four big miRNA data cohorts, with a total of more than 1,143 NPC patients, we develop and validate a five-microRNA prognostic signature. It could accurately predict the prognosis of patients with NPC and progress by constructing a nomogram, combined with risk score and N stage, to accurately predict the prognosis of patients with NPC. Besides this, based on the risk score, a potential layered treatment scheme for NPC was proposed.

## Data Availability Statement

The data of the TCGA database can be downloaded from UCSC Xena website (https://gdc.xenahubs.net). All the microarrays data (GSE32960, GSE36682, GSE70970) can be downloaded from the Gene Expression Omnibus database (GEO, https://www.ncbi.nlm.nih.gov/geo/).

## Author Contributions

SW and SL conceived and designed the study. SH involved in most of the revision of the manuscript. CZ and SW performed the analysis procedures. SW, SH, CZ, JX, and SL analyzed the results. SW, SH, JX, and SL contributed the analysis tools. SW, SH, CZ, JX, and SL contributed to the writing of the manuscript. All authors contributed to the article and approved the submitted version.

## Funding

This work was supported by the National Natural Science Foundation of China (81902781).

## Conflict of Interest

The authors declare that the research was conducted in the absence of any commercial or financial relationships that could be construed as a potential conflict of interest.

The reviewer YW declared a shared affiliation to the authors at the time of the review.

## Publisher’s Note

All claims expressed in this article are solely those of the authors and do not necessarily represent those of their affiliated organizations, or those of the publisher, the editors and the reviewers. Any product that may be evaluated in this article, or claim that may be made by its manufacturer, is not guaranteed or endorsed by the publisher.
