# Supplementary material for: A Five-MicroRNA Signature Predicts the Prognosis in Nasopharyngeal Carcinoma
Source: Front Oncol. 2021 Sep 9;11:723362. doi: 10.3389/fonc.2021.723362 (PMC8459682; doi:10.3389/fonc.2021.723362)
Supplement: Supplementary file 1 [file DataSheet_1.docx]

**Supplementary information**

**Supplementary figures**

**
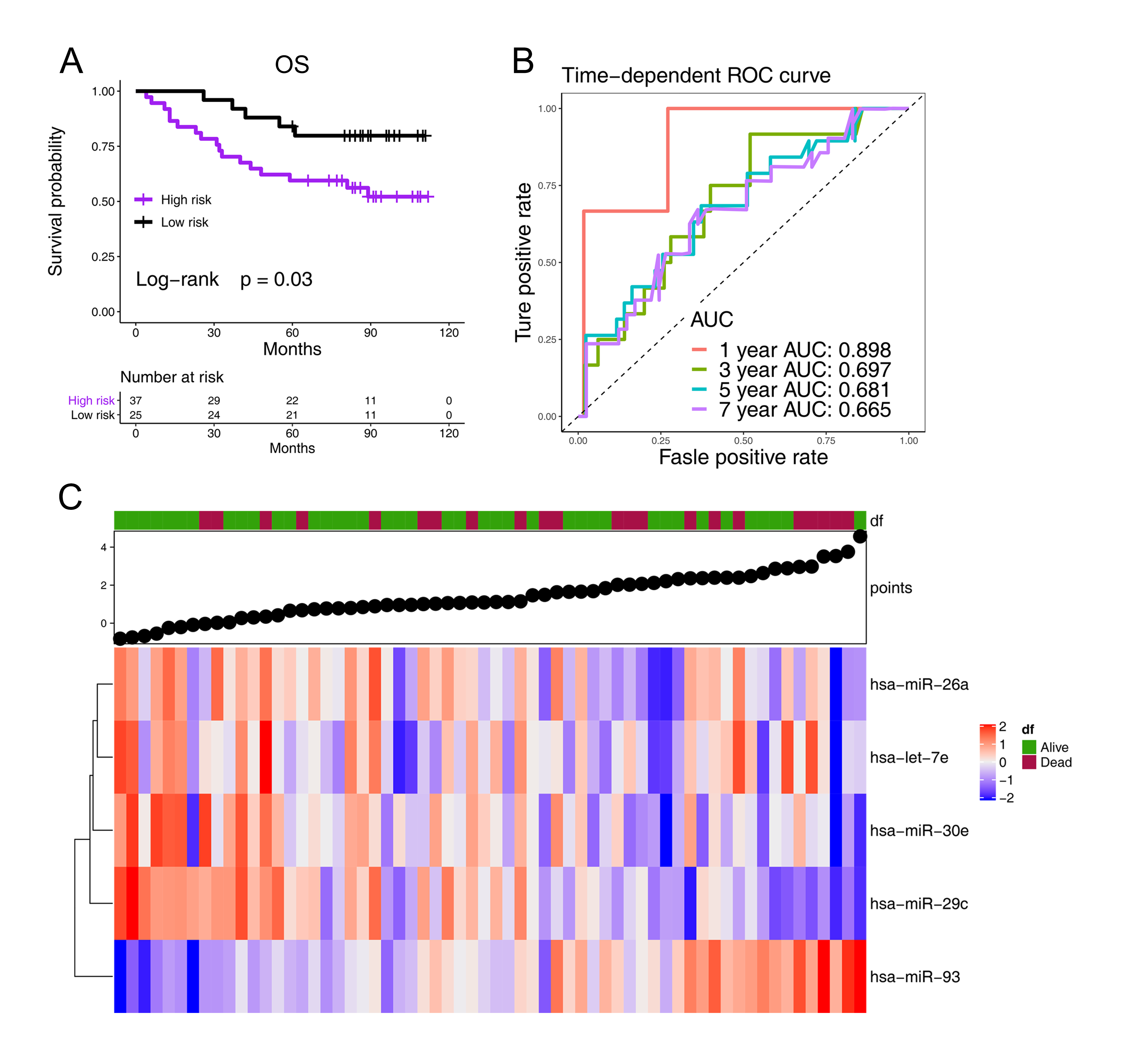
**

**Figure S1.** The prognostic value of 5-microRNA was validated in external validation cohort GSE36682. **(A)** The overall survival curve in the high- and low-risk group. **(B)** The time-dependent ROC of different risk group in GSE36682 cohort. **(C)** Heatmap of the expression of 5-microRNA in the external validation cohort GSE36682.


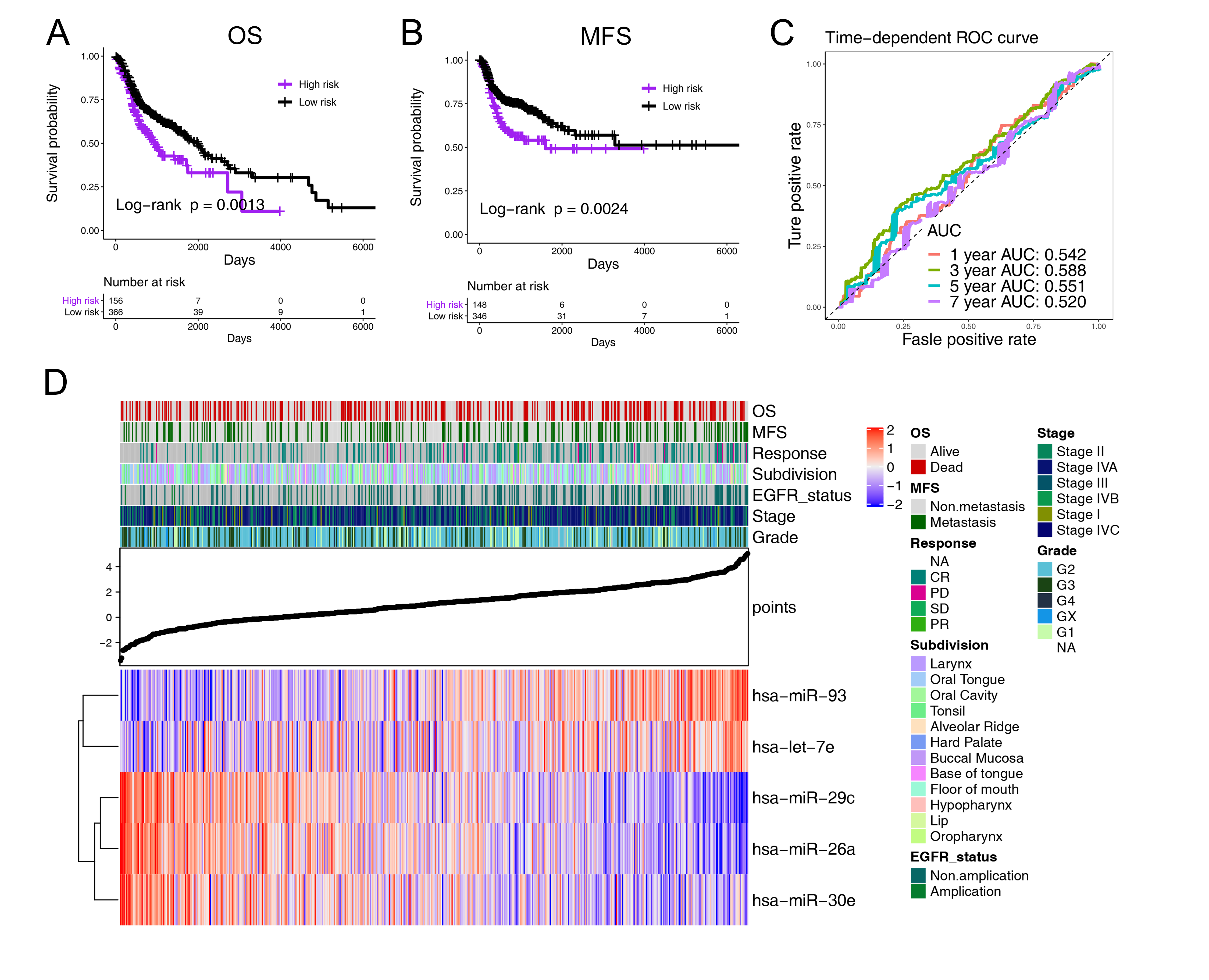


**Figure S2.** The prognostic value of 5-microRNA was validated in external validation cohort TCGA-HSSC. **(A)** The overall survival and **(B)** metastasis-free survival curve in the high- and low-risk group. **(C)** The time-dependent ROC of different risk group in TCGA-HSSC cohort. **(D)** Heatmap of the expression of 5-microRNA in the external validation cohort TCGA-HSSC.


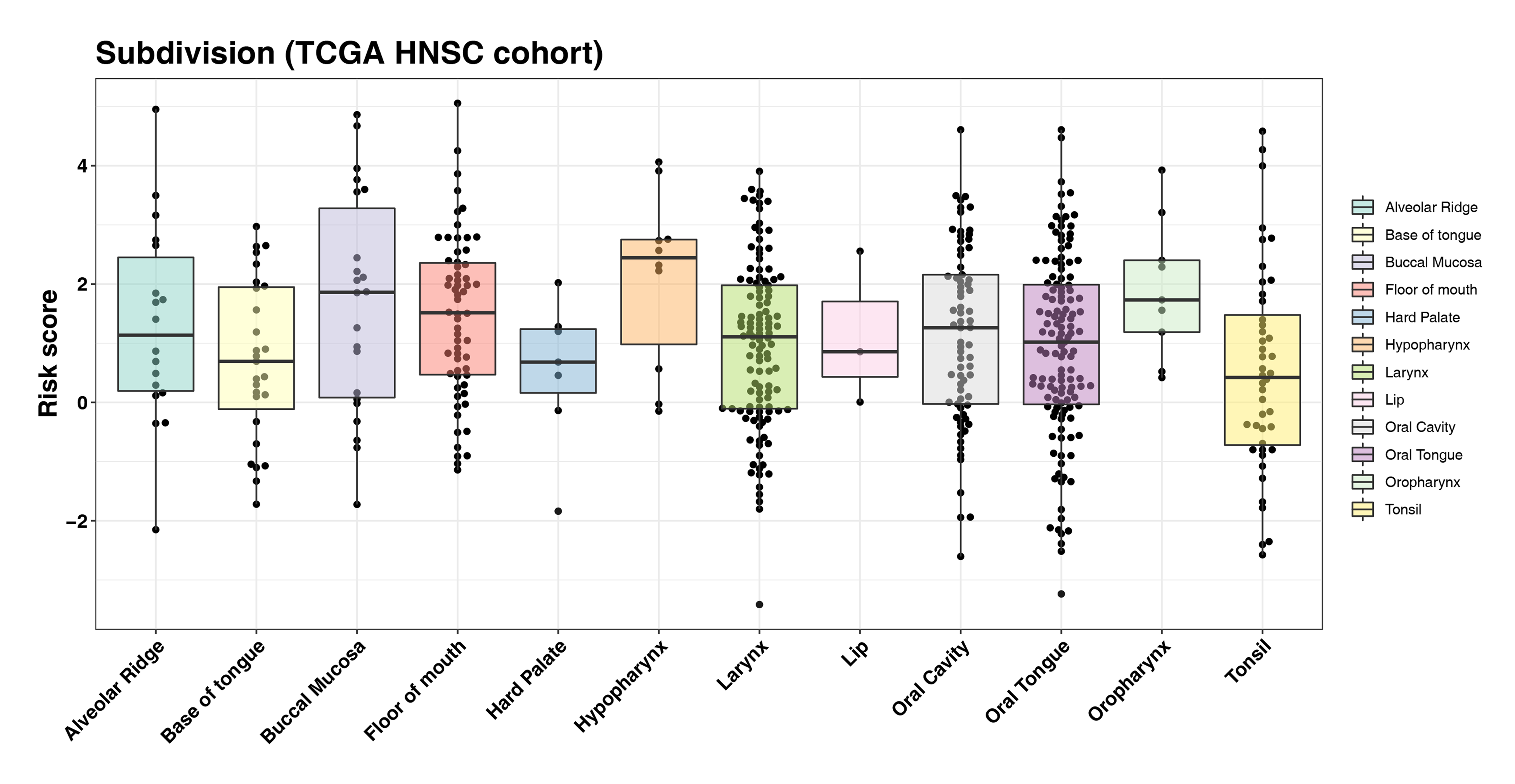


**Figure S3. The risk score distribution of subdivided anatomical sites in the TCGA-HNSC cohort.**

**
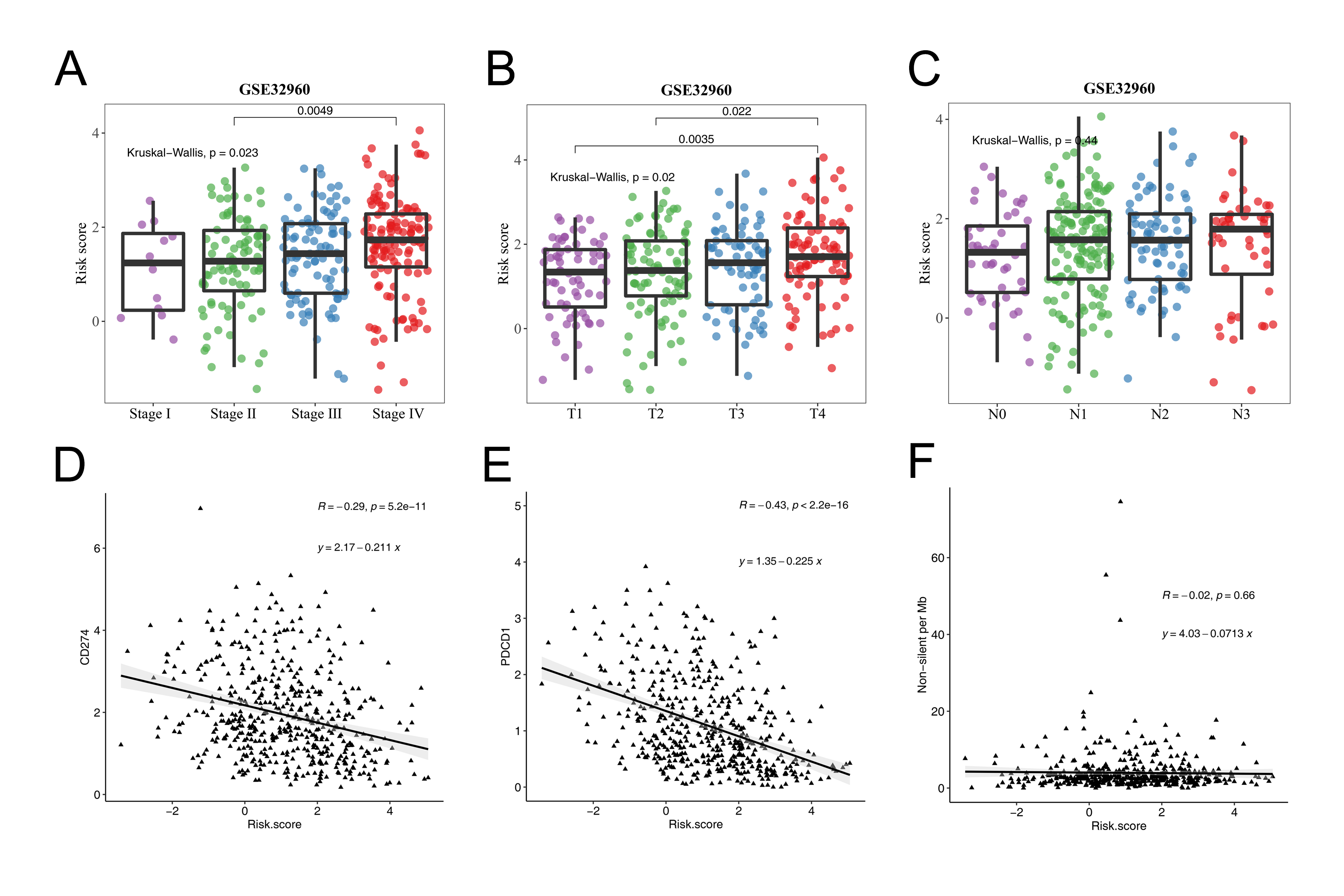
**

**Figure S4. Association analysis of risk score with clinical characteristics, immune checkpoint, and TMB.**
